# Invasive Versus Echocardiographic Aortic Valve Gradients Pre and Post Balloon-Expandable TAV-in-TAV for Failed TAVR Prosthesis

**DOI:** 10.1016/j.jacasi.2025.02.016

**Published:** 2025-04-29

**Authors:** Luai Madanat, Ivan D. Hanson, Ahmad Jabri, Brian Renard, Amr E. Abbas

**Affiliations:** a Department of Cardiovascular Medicine, William Beaumont University Hospital, Royal Oak, Michigan, USA; b Oakland University, William Beaumont School of Medicine, Rochester, Michigan, USA

**Keywords:** discordance, echocardiographic mean gradients, invasive transaortic mean gradient, TAV-in-TAV, transcatheter aortic valve replacement

Echocardiography is a well-established method for calculating transvalvular mean gradients (MGs) using transaortic velocities, particularly in cases of severe aortic stenosis and structural valve degeneration.^1^ Although it is widely used to evaluate bioprosthetic valve function, studies have highlighted notable discrepancies between echocardiographic and invasive measurements of transaortic mean gradients following both transcatheter aortic valve replacement (TAVR) and valve-in-valve TAVR for surgical valve deterioration.^2^^,^^3^ This phenomenon has led experts to caution against relying exclusively on echocardiography-derived gradients to assess the function of normal bioprosthetic valves. However, data on whether such discordance is present and its magnitude in patients undergoing TAVR-in-TAVR (TAV-in-TAV) for failed TAVR prostheses remain limited.

The aim of this study was to examine the discordance between echocardiography-derived transaortic mean gradients and invasive measurements in patients undergoing TAV-in-TAV for failed TAVR prostheses. We included consecutive patients with failed bioprosthetic TAVR prosthesis presenting for TAV-in-TAV procedure at our institution. The Institutional Review Board at Beaumont Health approved the study.

Invasive MG immediately above aortic valve leaflets were obtained using standardized invasive hemodynamics with 2 pigtails both pre– and post–TAV-in-TAV. Simultaneous echocardiographic MG was obtained in the standardized fashion by transthoracic echocardiography. Numerical values presented as mean ± SD, and paired Student's *t*-test was used to compare modalities. A 2-sided *P* value of <0.05 was considered statistically significant.

A total of 10 patients were included in our analysis. The mean age was 85 years, and 60% were women. The majority had failed self-expandable valves 7/10 (70%). Mechanism of valve failure was mixed aortic stenosis and regurgitation in 3 patients, while the remaining 7 patients had primarily severe aortic regurgitation. Of the 10 patients, 9 had a balloon expandable valve implanted at the time of TAV-in-TAV. Details of their baseline characteristics and valve types are included in Table 1.

**Table 1:** Baseline Demographic and Valve Characteristics

	Patient 1	Patient 2	Patient 3	Patient 4	Patient 5	Patient 6	Patient 7	Patient 8	Patient 9	Patient 10
Age, y	84	88	81	71	81	97	85	89	82	88
Sex	Female	Female	Male	Male	Male	Female	Male	Female	Female	Female
Ejection fraction, %	60	60	55	65	60	50	60	65	20	60
Original TAVR valve type	BE	BE	SE	SE	SE	SE	SE	SE	BE	SE
Original TAVR valve size, mm	23	23	29	29	34	34	29	29	23	34
Mechanism of valve failure	Mixed AS/AR	Mixed AS/AR	Primary AR	Primary AR	Primary AR	Primary AR	Primary AR	Primary AR	Mixed AS/AR	Primary AR
Pre–redo-TAVR echo MG, mm Hg	36	29	8	16	13	4	12	10	35	6
Pre–redo-TAVR invasive MG, mm Hg	34	23	8	23	12	1	8	2	40	2
Doppler velocity index	0.36	0.22	0.33	0.48	0.39	0.60	0.50	0.64	0.20	0.69
Redo-TAVR valve type	BE	SE	BE	BE	BE	BE	BE	BE	BE	BE
Redo-TAVR valve size, mm	23	26	26	23	29	26	23	23	20	26
Post redo-TAVR echo MG, mm Hg	16	11	4	11	2	6	9	8	12	2
Post redo-TAVR invasive MG, mm Hg	5	0	0	6	0	0	1	0	0	0

AR = aortic valve regurgitation; AS = aortic valve stenosis; BE = balloon-expandable; SE = self-expandable; TAVR = transcatheter aortic valve replacement.

Pre–TAV-in-TAV, echocardiographic MG (16.9 ± 12.0 mm Hg) were similar to invasive MG (15.3 ± 14.0 mm Hg; *P =* 0.305). However, following TAV-in-TAV, invasive MG (1.2 ± 2.3 mm Hg) was lower than immediate postprocedure echocardiographic MG (8.1 ± 4.6 mm Hg; *P* < 0.001) and predischarge echocardiographic MG (11.2 ± 4.4 mm Hg; *P* < 0.001). There was a trend toward higher echocardiographic pre-discharge MG compared with immediate postprocedure MG, however, did not reach statistical significance *(P =* 0.054) (Figure 1).

**Figure 1:**
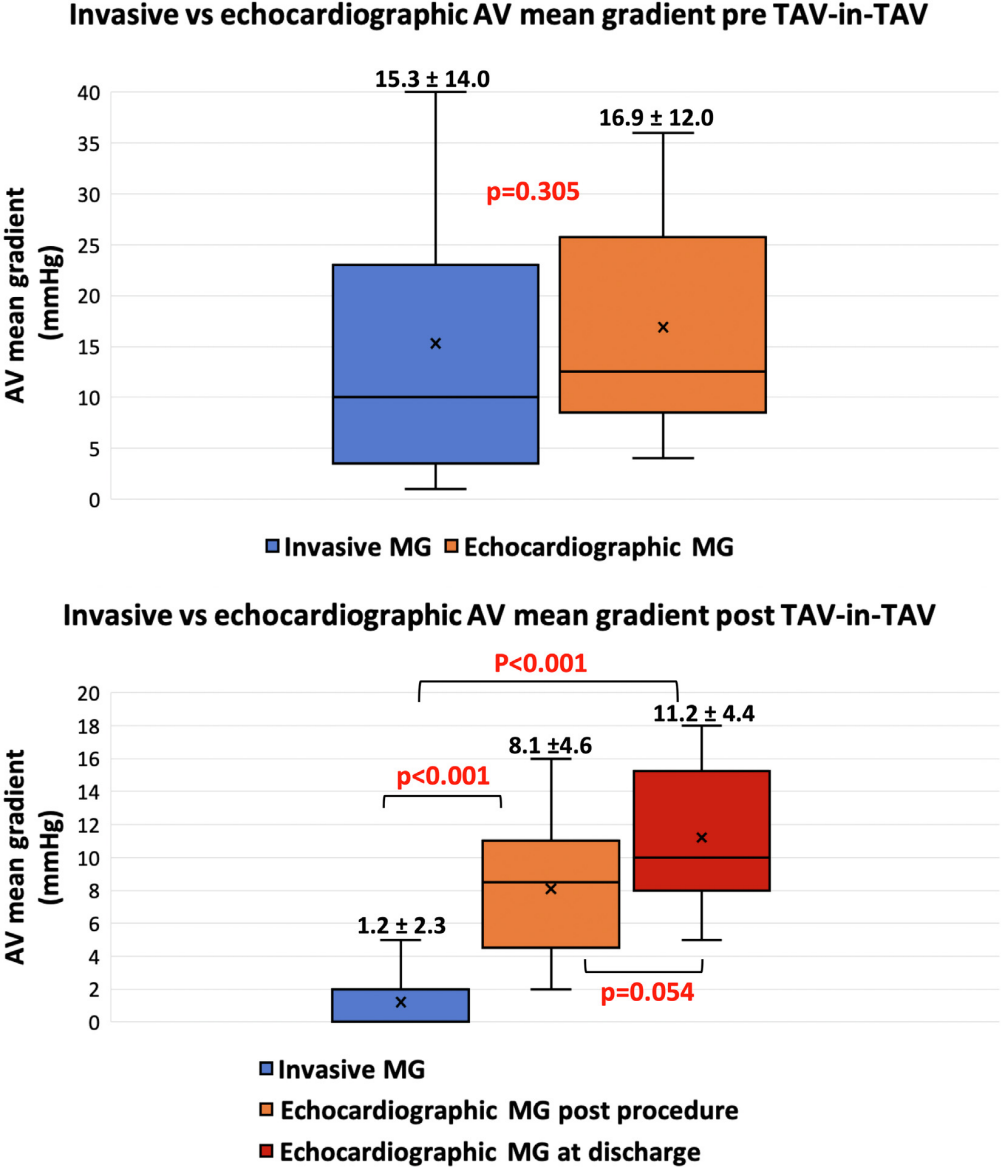
Box and Whisker Plot Comparing Echocardiographic and Invasive Mean Gradients Pre–transcatheter aortic valve replacement in transcatheter aortic valve replacement (TAV-in-TAV): Echocardiographic aortic valve mean gradients (MGs) (16.9 ± 12.0 mm Hg) were similar to invasive MGs (15.3 ± 14.0 mm Hg; *P =* 0.970). Post–TAV-in-TAV: Invasive aortic valve MG (1.2 ± 2.3 mm Hg) were lower than immediate postprocedure (8.1 ± 4.6 mm Hg; *P* < 0.001) and predischarge echocardiographic MGs (11.2 ± 4.4 mm Hg; *P* < 0.001), demonstrating the presence of discordance post–TAV-in-TAV. There was a trend toward higher echocardiographic predischarge MG compared with immediate postprocedure MG, however, did not reach statistical significance *(P =* 0.054).

None of the patients had any residual valvular or paravalvular regurgitation postprocedure.

This study demonstrates that echocardiographic and invasive MG correlate well in failed TAVR prostheses. However, following TAV-in-TAV, invasive MGs were consistently lower than both immediate postprocedure and predischarge echocardiographic MGs. In severe aortic stenosis, discordance between echocardiographic and invasive mean gradients is often attributed to pressure recovery—a phenomenon where pressure downstream from the aortic valve increases as kinetic energy partially reconverts to potential energy, thereby lowering the observed transvalvular gradient. However, post-TAVR, prior reports has shown that echocardiography may overestimate transaortic MG because of inherent limitations in the Bernoulli equation and noninvasive pressure recovery models when applied to normal functioning TAVR valves.^4^ Discordance between invasive and echocardiographic pressure gradients are more prevalent with balloon-expandable valves, particularly in smaller sizes.^3^ Among the 10 valves utilized in the TAV-in-TAV procedure in this study, only one was a self-expanding valve. Consequently, our findings cannot be generalized to all valve types. Moreover, the study's small sample size and retrospective nature pose challenges in drawing definitive conclusions, because they may lead to statistical limitations and a higher risk of bias. However, these findings underscore the importance of recognizing and accounting for this discordance when evaluating patients with bioprosthetic TAVR valves.

## Funding Support and Author Disclosures

Dr Hanson is a proctor for Edwards Life Sciences. Dr Abbas has received research grants and consulting fees from Edwards Life Sciences. All other authors have reported that they have no relationships relevant to the contents of this paper to disclose.
